# Spontaneous myogenic fasciculation associated with the lengthening of cardiac muscle in response to static preloading

**DOI:** 10.1038/s41598-021-94335-w

**Published:** 2021-07-20

**Authors:** Shouyan Fan, Lingfeng Gao, Annie Christel Bell, Joseph Akparibila Azure, Yang Wang

**Affiliations:** 1 Laboratory of Extreme Environment Sports Medicine, Hainan Medical University, No. 3 Chengxi Xueyuan Road, Haikou, Hainan PFTZ 571199 China; 2 School of Emergency Trauma, Hainan Medical University, Haikou, Hainan PFTZ 571199 China

**Keywords:** Cardiology, Cardiovascular biology, Heart development

## Abstract

Force enhancement is one kind of myogenic spontaneous fasciculation in lengthening preload striated muscles. In cardiac muscle, the role of this biomechanical event is not well established. The physiological passive property is an essential part for maintaining normal diastole in the heart. In excessive preload heart, force enhancement relative erratic passive properties may cause muscle decompensating, implicate in the development of diastolic dysfunction. In this study, the force enhancement occurrence in mouse cardiac papillary muscle was evaluated by a microstepping stretch method. The intracellular Ca^2+^ redistribution during occurrence of force enhancement was monitored in real-time by a Flou-3 (2 mM) indicator. The force enhancement amplitude, the enhancement of the prolongation time, and the tension–time integral were analyzed by myography. The results indicated that the force enhancement occurred immediately after active stretching and was rapidly enhanced during sustained static stretch. The presence of the force and the increase in the amplitude synchronized with the acquisition and immediate transfer of Ca^2+^ to adjacent fibres. In highly preloaded fibres, the enhancement exceeded the maximum passive tension (from 4.49 ± 0.43 N/mm^2^ to 6.20 ± 0.51 N/mm^2^). The occurrence of force enhancement were unstable in each static stretch. The increased enhancement amplitude combined with the reduced prolongation time to induce a reduction in the tension–time integral. We concluded that intracellular Ca^2+^-synchronized force enhancement is one kind of interruption event in excessive preload cardiac muscle. During the cardiac muscle in its passive relaxation period, the occurrence of this interruption affected the rhythmic stability of the cardiac relaxation cycle.

## Introduction

Myogenic control is the intrinsic autoregulation of cardiac rhythm and is considered to be the first sublevel in the control of cardiac function^1^. Myogenic control has been demonstrated in the autoregulation of the mesenteric, skeletal muscular, cerebral, renal, and coronary circulation systems but rarely in heart muscles. Over the past few years, a specific heart failure syndrome has been reported: takotsubo cardiomyopathy, which is characterized by the sudden acute, reversible enlargement of the left ventricle chamber so that it resembles an octopus trap when viewed by ventricular chamber imaging^2^. In this type of myopathy, the cardiac muscle developed extreme lengthening that initiated intrinsic myogenic autoregulation in the muscle. The development of muscle passive properties play the important role in myogenic control in this type of excessive preload heart. The erratic passive properties impede blood filling, and onset of blood ejection, is the key event that involved in biomechanical compensatory in takotsubo cardiomyopathy. Therefore, clarifying the role of force enhancement generation in excessive preload cardiac muscle is helpful for understanding the ventricular dysfunction in takotsubo cardiomyopathy.

The acute blood filling is one kind of active stretch, induce a lengthening preload in ventricular muscle. The muscle fibres are in a transient passive relaxation state after this stretch, but continuously tolerate the lengthening stress. It was frequently reported that myogenic spontaneous force enhancement easily occurred during tolerating sustained static stretch. When a skeletal muscle fibre instantaneously stretches beyond its optimum length, a significant myogenic twitch can be observed^3^. This spontaneous twitching is widely mentioned in studies of isolated striated muscle fibres^4^, muscle–tendon units^5^, flexor muscles^6, 7^, and skinned muscle fibres^8^. Spontaneous twitching is considered to be the mechanical response of sarcomere rearrangements and elastic properties in cardiac myofibrils^9^. Mechanical stretching elongate the muscle fibers, induces differences in sarcomere lengths, and as a result, increased recruitment of myosin heads occurs^10, 11^. In the lengthened muscle fibres, the intensity of the tension is sustained passively because concentrated Ca^2+^ binding alters calcium sensitivity in sarcomeric myofilaments^12, 13^, and Ca^2+^ binding to myosin-actin sites is not attenuated^12^. However, the perturbation of the dynamic distribution of intracellular Ca^2+^ ([Ca^2+^]_i_) in the lengthening tolerating muscle fibres have rarely been studied, and this may be related to the pathogenesis of takotsubo cardiomyopathy.

**Figure 1:**
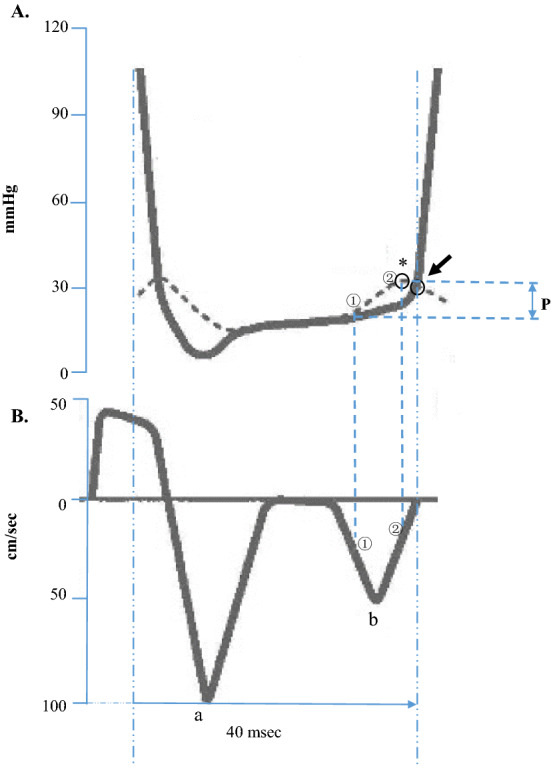
Illustration of the excessive load in the late diastole and the relative muscle relaxation velocity in cardiac ventricles. (**A**) Left ventricle pressure (solid curve) and left atrial pressure (dotted curve) in the ventricular diastolic phase. The areas marked ① and ② represent the rising atrial systolic pressure (kick from the atrium), and * is the maximum atrial systolic pressure. ② was the equilibrium point of atrial pressure to the moment ventricular pressure. The ventricle significantly contracted after the equilibrium point ②. Symbol P indicates the kick amplitude (excessive load amplitude) in the ventricle late diastole phase. (**B**) The ventricle muscle relaxation velocity in each diastole and systole cycle. The handstand peak wave a (the upside down of the peak wave) is the myocardial velocity during early filling, b is myocardial velocity during filling produced by atrial contraction. In handstand peak wave b, the curve between point ① and ② show the relaxation velocity response to ① and ②. The ventricular muscle velocity was significantly reduced because of excessive load from the kick. In takotsubo ventricle, massive volume filling interrupted the normal pattern of peak waves a and b. Myocardium was extremely overlengthened. The images originated from Zile and Brutsaert^16^ and were modified. All rights reserved.

In the conventional view of cardiac diastole, muscle relaxation is regarded as merely passive and an essential step in the cardiac cycle. The intracellular calcium decline, the thin filament deactivation, and the cross-bridge cycling kinetics are actively regulated^14^. However, cardiac diastole is responsive to the muscle passive properties. In the stages of one diastole, the ventricular muscle is in a passive relaxation state (diastasis, passive filling) after completed the active relaxation (suck the blood). This passive filling phase share approximately 71% of the total clinical diastolic phase. When the heart is in its late diastole (passive filling phase), cardiac ventricular wall tolerates excessive mechanical loads, which has been referred to as a “kick from the atrium”^15^. Figure 1A shows the variation of preload within left ventricle. Figure 1B shows Doppler tissue imaging-determined myocardial velocity in one complete cardiac cycle. This non-invasive method analysed muscle diastole and systole functions at the anterior wall or posterior wall of left ventricle. During the ventricular muscle was in the late diastole, a transient atrium pressure increasing which was induced by left atrium contraction in Fig. 1A (the dotted curve from ① and to ②, where ① is the inflection point of pressure increasing in left atrium, * is the maximum transient atrial pressure; the arrow marks the atrial equivalent point of the “kick from the atrium” to the ventricular diastole pressure)^16^, evoked a secondary left ventricle myocardial velocity variation in Fig. 1B. In Fig. 1B, the handstand peak waives are the myocardial velocity in left ventricle diastole. The descending limb means myocardial velocity increasing, while ascending limb is the reducing of the velocity. The upside down of the peak wave a is the myocardial velocity during early filling phase, wave b is myocardial velocity in the later filling phase. In wave b, the curve between point ① and ② represents the acceleration and deceleration of the stretched muscle during the “kick from atrium”. In takotsubo syndrome, the enlarged ventricle chamber created the aberrated waveforms in a and b. This is because of the left ventricular diastole formed a 72 mmHg intraventricular pressure gradient (4.6 mmHg in normal ventricle)^17^. The ventricle increased pressure gradient was because of the excessive volume filling^18^. This excessive preload intensified ventricular muscle passive properties. Ventricular muscle passive relaxation and onset of systole were interrupted by such erratic passive properties. Therefore, clarifying the variation of passive properties in over lengthened myocardium is a critical step for understanding the weakened ventricle function in takotsubo cardiomyopathy.

In this study, we hypothesize: (1) Cardiac muscle generate a visible enhancement of myogenic spontaneous force (force enhancement, FE) during the muscle tolerating the sustained static stretch, (2) The force enhancement generation may not in a steadily pattern under the excessive preload. If so, the variation force enhancement will interrupt the cardiac muscle relaxation process. To testify this hypothesis, we verified the steadily growing pattern of FE under the stepping lengthening preload in cardiac muscle. How this myogenic spontaneous force event contribute to erratic passive property, further interrupt the normal passive relaxation in cardiac muscle. We designed a rapid active stretching protocol that mimics the muscle passive tension variation in dilated ventricle walls. We prepared intact cardiac muscle fibres (trabeculae bundle) to understand passive tension relative myogenic force enhancement while the cardiac muscle underwent sustained static lengthening. The lengthened muscle [Ca^2+^]_i_ redistribution was evaluated. The muscle membrane integrity is considered as an effective compound in muscle tension processing. The destructed membrane may increase ionic permeability, increasing cross membrane transportation, influence muscle passive properties. Therefore, we prepared shed cardiac muscle fibres that was skinned by pore-forming protein *CfTX-1.* The shed cardiac muscle membrane was partially destructed^19^. The passive properties was compared between the intact and shed preparations. Through the above laboratory studies we expect to verify the FE relative erratic passive property in excessive preload cardiac muscle. This is helpful for clarifying the excessive preload induced transient ventricular akinesia in takotsubo cardiomyopathy.

## Results

### The force enhancement occurrence in intact muscle fibres

**Figure 2:**
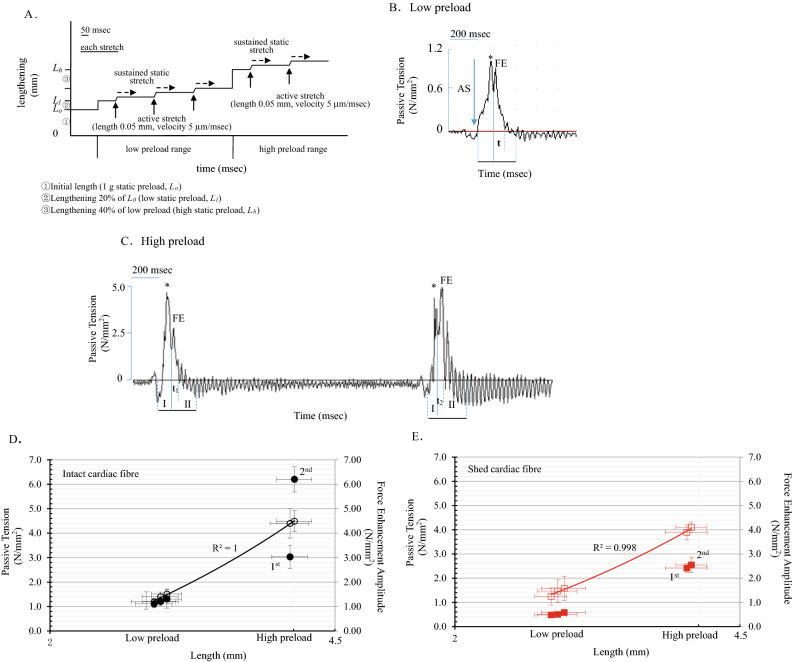
Real-time occurrence of force enhancement in excessive preload cardiac muscle fibres. (**A**) Sketched schematic of stretching cardiac papillary muscle fibres. The fibres were first lengthened to obtain a 1 g initial preload (① in the y-axis, *L*_*o*_). The fibres were further slowly lengthened to 20% of their initial preloaded length (lengthening ② in y-axis), and this length was defined as the low preload (*L*_*l*_). The active stretch was operated under *L*_*l*_. After the low preload tests, the fibres further slowly lengthened to 40% of the final lengthening (③ in y-axis) to obtain the high preload (*L*_*h*_). The fibres were actively stretched twice under *L*_*h*_. The dotted line with the arrow indicates that the fibre tolerated sustained static stretch. (**B**) The myograph of stretches in cardiac fibre. When the fibre bears *L*_*l*_, an active stretch induces an increase in passive tension (AS and on time scale I; *, peak of passive tension, PT_max_). Subsequently, a force enhancement occurred (FE, peak of force enhancement; t, force enhancement prolonging time). The fibre tolerated sustained static stretching during this period (II, the attenuation of passive tension). EF amplitude (A_FE_) normally did not surpass its PT_max_. The stretch repeated three times under the *L*_*l*_ condition. (**C**) In *L*_*h*_cardiac fibre, the 1st and 2nd A_FE_ dramatically increased, furthermore the 2nd A_FE_ significantly surpassed its PT_max_. (**D**) PT_max_ presented a perfect linear correlation throughout preload range in intact cardiac fibres (R^2^ = 0.99, n = 10). The A_FE_ significantly increased in *L*_*h*_cardiac fibres. Furthermore, the 2nd A_FE_ surpassed its PT_max_. The erratic occurrence of force enhancement was observed in high preload cardiac fibre. (**E**) PT_max_ presented a perfect linear correlation throughout preload range in shed cardiac fibres (R^2^ = 0.99, n = 10). The A_FE_ reduced in *L*_*h*_ cardiac fibres. No surpassing of its PT_max_ observed.

Figure 2A is the schematic diagram of mechanical stretch. Figure 2B shows the passive tension waveform in the low preload range, which included a passive tension peak (I active stretch on this time scale, * passive tension amplitude peak) and subsequent passive tension attenuated after the peak (II, passive tension attenuation on this time scale). The force enhancement caused passive tension in its attenuation period (FE in Fig. 2B). Figure 2C shows the typical force enhancement waveform in the high preload range (resembles the excessive preload). PT_max_ had a linear correlation throughout the whole preload range; however, the force enhancement amplitude A_FE_ was not correlated.

The stretched force enhancement waveform values in the low preload range are summarized in Table 1. Symbol t defined as the force enhancement prolonged time. The 1st stretched PT_max_ was 1.19 ± 0.14 N/mm^2^, force enhancement amplitude was A_FE_ 1.11 ± 0.16 N/mm^2^, t was 123.72 ± 4.58 ms, and the total attenuation (II) was 216.51 ± 9.44 ms. Therefore, t possessed 57.14% of the relaxation on this time scale.

**Table 1:** The active stretched passive tension and relative force enhancement in low preload cardiac muscle fibres.

Preloading condition	Preparation	Stretch	PT_max_ (N/mm^2^)	A_FE_ (N/mm^2^)	FE prolonged time	
					t (ms)	% of II
Low preload range	Intact muscle (n = 10)	1st	1.19 ± 0.14	1.11 ± 0.16	123.72 ± 4.58	57.14
		2nd	1.40 ± 0.19	1.21 ± 0.17	132.51 ± 4.51	69.23
		3rd	1.51 ± 0.21	1.31 ± 0.37	141.20 ± 5.33	83.33
	Shed muscle (n = 10)	1st	1.25 ± 0.35	0.48 ± 0.03***	126.88 ± 6.79	72.73
		2nd	1.47 ± 0.46	0.50 ± 0.04***	130.89 ± 5.41	53.94
		3rd	1.58 ± 0.50	0.58 ± 0.09***	138.17 ± 7.61	44.44

The passive tension peak PT_max_ and the force enhancement amplitude A_FE_ are summarized in Fig. 2D, wherein the A_FE_ never surpassed its PT_max_. PT_max_ presented a perfect linear correlation throughout the whole preload range (Fig. 2D, PT_max_, black hollow circle, R^2^ = 0.99), however nonlinear relationship of A_FE_ in this ranges.

As summarized in Table 2, in the high preload range, the force enhancement was unstable during muscle stretching. A_FE_ did not fit the linear relationship because of the scatter of the amplitude (Fig. 2D solid dots). In the greatest stretch (2nd stretch), the force enhancement surpassed the passive tension; therefore, it significantly disrupted the passive tension during lengthening tolerance (2nd PT_max_ and AFE were 4.49 ± 0.43 N/mm^2^ and 6.20 ± 0.51 N/mm^2^, ***p < 0.001 respe*c*tively in Table 2).

**Table 2:** The active stretched passive tension and relative force enhancement in excessive preload cardiac muscle fibres.

Preloading condition	Preparation	Stretch	PT_max_ (N/mm^2^)	A_FE_ (N/mm^2^)	FE prolonged time	
					t (ms)	% of II
High preload range	Intact muscle (n = 10)	1st	4.40 ± 0.60	3.03 ± 0.48	127.08 ± 8.31	65.82
		2nd	4.49 ± 0.43	6.20 ± 0.51***	117.42 ± 10.80	21.89
	Shed muscle (n = 10)	1st	3.89 ± 0.31	2.42 ± 0.19	105.29 ± 11.10	15.52
		2nd	4.11 ± 0.22	2.55 ± 0.31	99.20 ± 9.90	10.14

### The force enhancement occurrence in shed muscle fibres

PT_max_ presented a perfect linear correlation throughout the preload range (Fig. 2E, R^2^ = 0.99; hollow square, PT_max_). In the low preload range, the force enhancement amplitude A_FE_ significantly declined (Fig. 2E, black square). As shown in Table 1, A_FE_ significantly reduced in shed preparation in comparison to the intact muscle fibres (***p < 0.001).

In the high preload range, A_FE_ in 1st and 2nd did not surpass the respective PT_max_ in each stretch (2.42 ± 0.19 N/mm^2^ and 3.89 ± 0.31 N/mm^2^ in the 1st stretch, 2.55 ± 0.31 N/mm^2^ and 4.11 ± 0.22 N/mm^2^ in the 2nd stretch, respectively). The force enhancement extended time t was prolonged. There was no statistic difference between shed and intact preparations.

### The [Ca^2+^]_i_ redistribution in sustained static stretch muscle fibres

**Figure 3:**
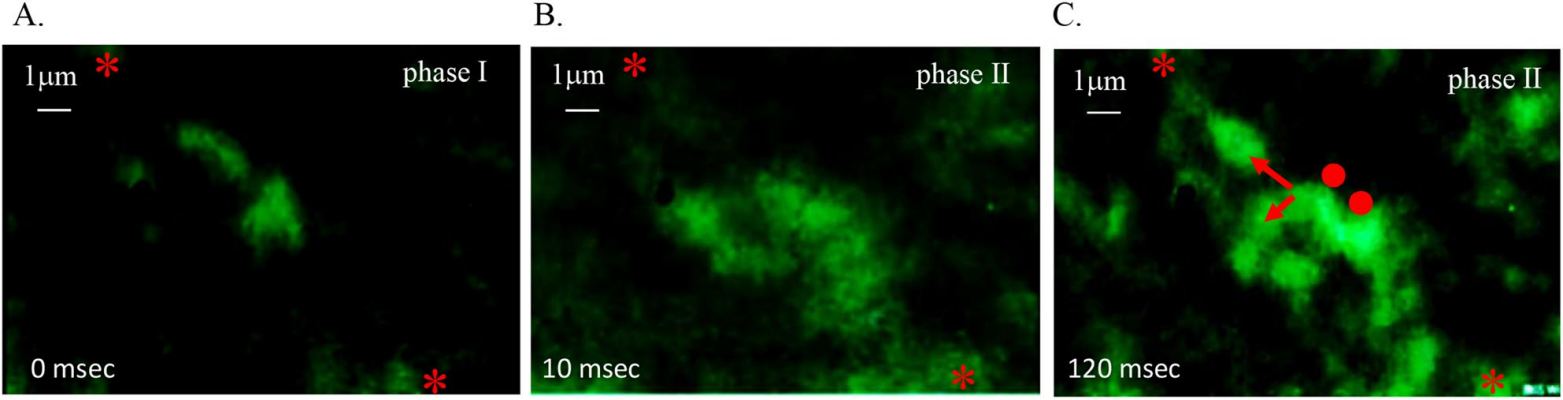
Real-time [Ca^2+^]_i_ image during tolerating sustained static stretch in excessive preload cardiac muscle fibres. (**A**) Fluorescence imaging of [Ca^2+^]_i_ in *L*_*l*_ muscle fibres before active stretching. The space between * was the [Ca^2+^]_i_ redistribution observation area during tolerating sustained static stretch. (**B**) Fluorescence imaging of [Ca^2+^]_i_ primary recruitment after one active stretch. On the myograph, the passive tension is on its attenuation phase. [Ca^2+^]_i_ rapidly recruited in some ultrastructure area in steady state muscle fibres. (**C**) Fluorescence imaging of [Ca^2+^]_i_ secondary recruitment and transmission after one active stretch. On the myograph, force enhancement occurred at this time. [Ca^2+^]_i_ rapidly transmitted to the adjacent ultrastructure area. Dots are the [Ca^2+^]_i_ extremely activated area, and arrows are the dimension of [Ca^2+^]_i_ transmission.

Figure 3A is a fluorescence image of [Ca^2+^]_i_ before active stretching. [Ca^2+^]_i_ showed no changes during the static preload; however, [Ca^2+^]_i_ redistribution immediately occurred after active stretching (Fig. 3B, the space between the * marks demonstrate the visible [Ca^2+^]_i_ fluorescence image area). After active stretching, [Ca^2+^]_i_ were rapidly diffused to the adjacent ultrastructure area. The [Ca^2+^]_i_ transmitted between the ultrastructural areas (Fig. 3C, the area between the asterisks symbol was the [Ca^2+^]_i_ redistribution observation area; the dot marked area was [Ca^2+^]_i_ extremely activated area; the arrow indicates the direction of [Ca^2+^]_i_ transmission) (Supplementary Video).

### The force enhancement tension-time integral TTI in cardiac muscle fibres

#### TTI in intact muscle fibres

**Figure 4:**
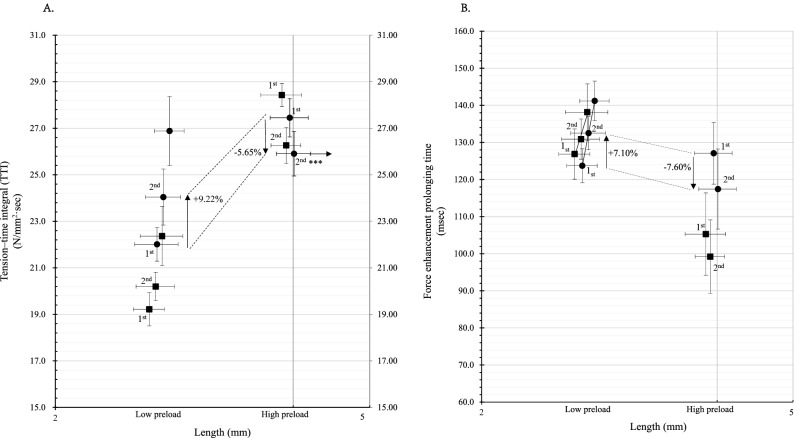
Force enhancement tension-time integral in excessive preload cardiac fibres. (**A**) The tension–time integral (TTI) of the force enhancement throughout the preload range of cardiac fibres. In intact fibres (black dots, n = 10), the TTI was significantly increased in the high preload range; however, the 2nd TTI was significantly reduced (5.65%) because of the shortening of the prolonging time (t_2_). These differences presented in shed fibres as well (n = 10). However, in low preload fibres, the adjacent stretch induced an increase in TTI increasing (3rd 10.57% increase). (**B**) The force enhancement prolonging time throughout the preload range of cardiac fibres. In intact fibres (black dots), t_2_ was significantly shortened in the high preload range (5.78% shortened, n = 10); however, in the low preload range, the last stretch-induced t was extended (6.56% increased, black dots, n = 10). These differences presented in shed fibres as well (black square, n = 10).

In the low preload range, the force enhancement tension-time integral TTI were 22.01 ± 0.73 N/mm^2^ s, 24.04 ± 1.21 N/mm^2^ s and 26.88 ± 1.48 N/mm^2^ s in the 1st, 2nd and 3rd stretches, respectively. In high preload range, TTI was 27.45 ± 0.84 N/mm^2^ s and 25.90 ± 0.95 N/mm^2^ s in 1st and 2nd stretch respectively; in fact, TTI in 2nd increased 9.22% of 1st in low preload range, but it reduced 5.65% of 1st in high preload range (Fig. 4A, solid dots tagged as 1st and 2nd in high preload range, *** means significant reduced, *p* < 0.01).

The force enhancement extended time (t) was prolonged in the low preload range, t_2_ prolonged 7.10% of t_1_; however t_2_ reduced 7.60% of t_1_ in high range (Fig. 4B, solid dots tagged 1st and 2nd). In high preload range, t_1_ shared 65.82% of II period, while t_2_ shared 21.89% (it was 57.14% and 69.32% respectively in low preload), which indicated the instability of the force enhancement occurrence in excessive preload cardiac muscle fibres.

#### TTI in shed muscle fibres

In the low preload range, TTI was reduced in comparison to the intact muscle fibres (19.22 ± 0.72 N/mm^2^ s, 20.20 ± 0.61 N/mm^2^ s and 22.36 ± 1.27 N/mm^2^ s in 1st, 2nd and 3rd stretch in shed preparation, the statistically significant *p* < 0.01). In the high preload range, TTI was 28.43 ± 0.49 N/mm^2^ s and 26.26 ± 0.78 N/mm^2^ s in 1st and 2nd stretch respectively (Fig. 4A, solid square in the high preload range). TTI in 2nd reduced 7.63% of 1st, however, it was 5.65% in intact muscle).

In low preload range, the force enhancement extended time (t) was prolonged after each active stretch; however, because the II was extended in high preload range, the % of t_1_ and t_2_ in II period was reduced (Table 1, 15.52% and 10.14% respectively).

### The role of CfTX-1 peptide

The *CfTX-1* peptide sequence is first identified in the tentacles of local *Aurelia aurita*.

In brief, frozen *A. aurita* tentacles were placed in an autolysis solution at + 4 °C overnight. After centrifuging at 20,000×*g* for 1 h at 4 °C, the resulting supernatant was immediately frozen at − 80 °C in a condenser chamber and vacuumed to extreme dryness (VirTis Bench Top freeze dryer, SP industries, Inc., PA, U.S.A..). The lyophilized crude venom was analysed on a 10% polyacrylamide gel. The *CfTX-1* peptide sequence was identified from the 43 kDa band. It was an 11 amino acid polypeptide that had a strong overlap with the positive strain of amino acid sequences 304–314 (*IFNFFDLmKVK*) of *CfTX-1* (UniProtKB-A7 L035). These *CfTX-1* 11 amino acids were further synthesized by a commercial solid-phase resin method, then lyophilized and analysed by HPLC and electrospray ionization tandem mass spectrometry (ESI–MS).

The synthesized *CfTX-1 peptide* is the effective membrane pore architecture which increased the ionic permeability, induced hyperpolarization in the urothelial membrane in vitro^20^, and improved mouse cardiac diastole in vivo.

## Discussion

Takotsubo cardiomyopathy was first described in 1980s that is an acute but often reversible left ventricular dysfunction. The reverse takotsubo cardiomyopathy is a variant characterized by the basal hypokinesis associated with apical hyperkinesis that resolves spontaneously. The cardiac magnetic resonance images presents a typical abnormal dilation of cardiac ventricles. During fulminant COVID-19, there were increasing numbers of cases of reverse takotsubo cardiomyopathy associated with the infection^21–23^ and epidemiological reports of 1.5% takotsubo cardiomyopathy in COVID-19 patients^24^ that prove the urgency and demands for understanding the mechanism of cardiac muscle akinesia. The force enhancement, that is an important biomechanical event in cardiac muscle, swing the passive properties, interrupted the diastolic relaxation cycle in excessive preload cardiac muscle, has important relevance to solving the mystery of midventricular akinesia in hearts with takotsubo cardiomyopathy.

In the 1950s, Abbott et al. first mentioned the occurrence of an excessive tension in 1.9 mm/s stretched toad sartorius^25^. Hill et al. reported an enhanced energy release and a transient increase in tension when toad sartorii were stretched and elongated by 5 mm within 393 ms^26^. Decades later, Sugi’s experiment showed semitendinosus fibre tension rising to the initial isometric value after falling below the initial isometric level at the end of an 80–150 cm/s stretch (0.8–1.5 mm/ms) and a delayed transient tension increase during a 30–60 cm/s (0.3–0.6 mm/ms) stretch^27^. These reports validated the relation between mechanical lengthening velocity and the occurrence of force enhancement in striated muscle. In this study, we used a software-controlled microstepping motor to obtain a stable mechanical lengthening velocity for each active stretch. A velocity 5 μm/ms was the condition for testing force enhancement development in intact and shed cardiac muscle fibres. A similar experimental method was used in the experiments of Edman, who was aware of the residual force enhancement from the nonuniform distribution of the myofilament during the length change^28^. The force enhancement corresponded to the magnitude of the stretch was gradually revealed in recent studies^29^. This correspondence was because of muscle fibre half-sarcomere nonuniformities and the sarcomeric component that was associated with Ca^2+^-induced stiffness^30^. In addition, force enhancement did not increase with stretch amplitude on the ascending branch, but on the descending branch of the force–length relationship^31^. From the above summarized results, we proves the existence of force enhancement in cardiac muscle fibres with increasing preloads. It can be concluded that force enhancement occurrence is more significant and variable in muscle fibres with preload, and this enhancement originated from the reduced myofibril cross-bridge interaction but affected by integrity of the membrane. Furthermore, we found variable occurrence of force enhancement in excessive preload cardiac muscle. It becomes the main component of the erratic passive properties, interrupts the cardiac muscle passive relaxation process. The interruptions are mainly manifested in: (1) unrestricted enhanced force amplitude; (2) erratic extension time of force enhancement; and (3) the flash redistribution of intracellular Ca^2+^ signaling in sustained static lengthened cardiac muscle.

TTI is the index for evaluating the time dependence of muscle tension. This index has been used to estimate the muscle energy output in isometric contractions^32^, to determine the capacity ratio of the diaphragm^33^, to analyse the transient contractions^34^, and to evaluate the effect*s* of contractile filament mutations on muscle twitches^35^. In cardiac muscle, TTI quantifies the tension development during the diastolic-systolic cycle^36, 37^. In this study, the results suggest that TTI was in a precarious state in high preload cardiac fibres, which reflected the force enhancement variation and the erratic passive properties in excessive preload cardiac muscle. It has been reported that TTI reflects calcium sensitivity interruption during tension development^38^, which is an index of fatigue development^39^. In this study, TTI precarious state in high preload range (TTI in 1st and 2nd stretch) was combined with the disturbed fluctuations of intracellular Ca^2+^ during muscle tolerating sustained static stretch. This result suggests the coupling mechanism between intracellular Ca^2+^ redistribution, TTI and erratic passive properties in excessive preload cardiac muscle.

Intact cellular membranes integrity are another important factor in maintaining normal tension in cardiac muscle fibres. In this study, the cellular membrane were influenced by two factors: (1) mechanical stretch induced permeability increasing in intact preparations, and (2) the *CfTX-1* pore-formation increased membrane permeability in shed preparation. The mechanical stretch-activated ion channels currents have been recorded in most cardiomyocyte types, which result in rapid alterations of cardiac electrical activity. This effects on cellular electrophysiology depend on the effective stretch target channels, stretch timing, and stretch characteristics (rate-of-rise and amplitude). In passive relaxed cardiac muscle, a sufficient stretch amplitude causes changes of membrane potential. Muscle membrane gives rise to excitation. The myogenic force enhancement was generated by this membrane excitation. However, the membrane excitability and relative force enhancement parameters were decreased due to the membrane pore-formation by *CfTX-1* peptide. *Chironex fleckeri* toxin (*CfTX*) has a homologous structure with three-domain Cry toxins (δ-endotoxins) in the N-terminal domain^40, 41^. *CfTX-1* act as the pore-forming toxin, have ion channel modulating characteristics. *CfTX-1* skinned cardiac muscle membrane disrupting normal transmembrane ion concentration gradients in cardiomyocyte*,* accelerate the first temporal derivative of the isometric contraction (dP/dt)^42^. Its specific lipid-dependent cell penetration induces transient membrane leakage for cardiomyocyte interactions at the atomic level, is involved in isometric force^43^, contributes to intracellular Ca^2+^ release, and improves cAMP and PKA activity in cardiomyocytes^44^. *Chironex fleckeri* toxin was reported leading to large increased intracellular calcium influx which caused spontaneous contractions, a decrease in developed force and an increase in resting force^45^. *Chironex fleckeri* venom effectiveness in cardiac papillary muscles was through Na^+^/Ca^2+^ exchanger and increasing influx of Ca^2+^. The mechanism was due to nonspecific membrane damage given the effects appeared quickly after venom exposure. Based on the previous experimental results, our results suggest that *CfTX-1* peptide pore-formation mainly reduce the generation of active tension, but not significant in passive tension reduction in excessive preload cardiac muscle. The results indicated that *CfTX-1* reduced the force enhancement during tolerating sustained stretch in excessive preload cardiac muscle. The passive properties relative parameter, such as force enhancement extension time (t) and TTI was also decreased. This evidence leads us to consider that the defective membrane-induced midventricular akinesia in takotsubo cardiomyopathy was originate from the membrane high permeability.

## Methods

### Mice cardiac papillary muscle fibre preparation

This animal experiment was reviewed and approved by the Hainan medical university institutional ethics board. Animal care was performed according to the ethical principles of the Guide for the Care and Use of Laboratory Animals (8th Edition, International Standard Book Number-13: 978-0-309-15401-7). All procedures performed conformed to the guidelines from directive 2010/63/EU of the European Parliament on the protection of animals used for scientific purposes. The animal study was conducted according to ARRIVE guidelines.

After the mice were anaesthetized with 3% pentobarbital sodium, intraperitoneal injection (0.1 ml/15 g body weight) was performed. Anaesthetic depth was monitored by disappearance of the corneal reflex. The physical method of cervical dislocation was used for mouse euthanasia.

The cardiac papillary muscles strips were isolated from the *Kunming* mice left ventricle (n = 10, male, 4 weeks, SPF grade). Cardiac papillary muscle strips were isolated from the *Kunming* mouse left ventricles (n = 10, male, 4 weeks, SPF grade). Muscle bundles were placed on a tick-marked slide, blunt isolated to 0.5 mm diameter by a top sharpened glass probe under dissection binocular microscope. This glass probe isolation method can ensure intact muscle fibre specimens are obtained. For shed preparation, fibres were separated from identical papillary bundle. Fibres were suspended in synthesized 35 mmol *CfTX-1* peptide solution (peptide sequence *IFNFFDLmKVK*), at + 4 °C for 5 min. All fibres were finally stabilized in *Ringer’s* solution before the tolerance tests. This biological shed method increased the membrane ionic permeability in resting muscle fibres.

For understanding cardiac muscle preparation passive properties during tolerating sustained static stretch, muscle preparation tolerance test was designed under the low preload and high preload conditions. The tolerance test performed on a stepper motor driven roller screw platform. One end of the cardiac fibre was fixed to the glass probe, which was fastened to a roller screw module; the other end was hooked on the reed of a Wheatstone bridge-type piezoelectric strain sensor (Model number JH-2 10 g, Beijing aerospace medical engineering institute, Beijing China). The fibres were kept horizontal on a + 4 °C chilled glass slide. The series of mechanical stretches was performed by a stepper motor, which was controlled by an Arduino Uno R3 board (Arduino, Allchips Ltd., Hong Kong). To obtain steady mechanical lengthening in each active stretch, the extent of the stretch was determined by the rotating speed and the angles of the stepper motor shaft, which was driven by the programmed pulse frequency of the Arduino board. Cardiac fibres tolerated 0.05 mm, 5 μm/ms linear lengthening in each active stretch.

### Muscle mechanical stretch and the myograph analysis

As the schematic diagram of mechanical stretch shows in Fig. 2A, slack cardiac fibres were slowly lengthened to remain taut in response to a 1 g preload was applied (Fig. 2A, ① on Y axis). The fibre length under this 1 g load was defined as the initial length (*L*_0_). The initially stretched fibres were stabilized on a glass slide and suspended in Ringer’s solution before tests.

The fibres were slowly lengthened by 20% of their *L*_*0*_ with a micro tuner to provide a static preload (Fig. 2A, ② on the Y-axis). This length was defined as the low preload (*L*_*l*_). The low preload fibres underwent rapid transient lengthening (0.05 mm lengthening with a velocity of 5 μm/ms), which was defined as active stretching (Fig. 2A, solid line arrow). The fibres subsequently tolerated static lengthening, which was defined as sustained static stretch (Fig. 2A, dotted arrow). The active stretch was repeated three times. After that, fibres were further lengthened by 40% to obtain a higher static preload (*L*_*h*_). The high preload fibres obtained active stretch twice. The passive tension trajectories (myograph) of the fibres were recorded by BL-420s data acquisition and analysis system (Chengdu TME technology Co. Ltd., Chengdu, China).

The passive tension maximum amplitude (PT_max_), the myogenic spontaneous force enhancement amplitude (A_FE_), the prolonging time (t), and the myogenic spontaneous force tension–time integral (TTI) were processed and calculated by TM_WAVE software (Chengdu techman software Co., Ltd., Chengdu China).

### Real-time visualization of [Ca^2+^]_i_ movement

Mechanical stretch-induced cardiac fibre Ca^2+^ ([Ca^2+^]_i_) movement was visualized in real time by fluorescence excitation. Before the lengthening tests, fibres were suspended on a support glass slide and incubated in 2 mM Flou-3 AM/DMSO solution for 20 min. The incubated fibres were excited by a 499 nm LED light beam to obtain 528 nm emission wavelength luminescence. Refraction fluorescence imaging of the spatial dynamics of [Ca^2+^]_i_ in stretching fibres was captured by an inversion microscope system (XDS-1B, Chongqing COIC industrial Co., Ltd. Chongqing China), and photographed by a 1200 TVL resolution camera connected to a high-speed analogue video system (SV2000E video capture system, Tairong Technology, Xuzhou, Jiangsu, China). The light beam excited a bright fluorescence region indicating activation of [Ca^2+^]_i_ in stretching fibres. To reduce the phototoxicity and dye bleaching to a minimum, the fibres were illuminated at low power. Activated [Ca^2+^]_i_ redistribution imaging was captured simultaneously myography. Because the fluorescence brightness was obtained from high-speed images in the suspended cardiac fibres, the absolute [Ca^2+^]_i_ concentrations could not be quantified by this method. However, real-time [Ca^2+^]_i_ qualitative analysis were performed for stretching fibres. The fluorescence images were processed via ImageJ (Ver. 1.53a, Wayne Rasband, NIH, U.S.A.).

### Statistical analysis

The values present as the mean ± SEM. The value differences under the preload conditions are analysed by using the two-factor without replication analysis (Excel 2013, 2012 Microsoft Corporation). *p* < 0.01 indicated a significant difference.

## Supplementary Information

Supplementary Video.

## Data Availability

The data that support the findings of this study are openly available in figshare at http://doi.org/10.6084/m9.figshare.14058197.

## References

[1] Nozdrachev AD (2005). A view of the cardiac rhythm control: Intrinsic regulation. Hum. Physiol..

[2] Dupliakov DV (2004). Ball-shaped spherical dilation of the left ventricular apex or "takotsubo" cardiomyopathy. Kardiologiia.

[3] Edman KA, Flitney FW (1978). Non-uniform behaviour of sarcomeres during isometric relaxation of skeletal muscle. J. Physiol..

[4] Noble MI (1992). Enhancement of mechanical performance of striated muscle by stretch during contraction. Exp. Physiol..

[5] Mahmood S, Sawatsky A, Herzog W (2021). Increased force following muscle stretching and simultaneous fibre shortening: Residual force enhancement or force depression—That is the question?. J. Biomech..

[6] Fortuna R, Power GA, Mende E, Seiberl W, Herzog W (2016). Residual force enhancement following shortening is speed-dependent. Sci. Rep..

[7] Hahn D, Riedel TN (2018). Residual force enhancement contributes to increased performance during stretch-shortening cycles of human plantar flexor muscles in vivo. J. Biomech..

[8] Fukutani A, Joumaa V, Herzog W (2017). Influence of residual force enhancement and elongation of attached cross-bridges on stretch-shortening cycle in skinned muscle fibers. Physiol. Rep..

[9] Kulke M (2001). Interaction between PEVK-titin and actin filaments: Origin of a viscous force component in cardiac myofibrils. Circ. Res..

[10] Brown LM, Hill L (1991). Some observations on variations in filament overlap in tetanized muscle fibres and fibres stretched during a tetanus, detected in the electron microscope after rapid fixation. J. Muscle Res. Cell Motil..

[11] Campbell KS, Janssen PML, Campbell SG (2018). Force-dependent recruitment from the myosin off state contributes to length-dependent activation. Biophys. J..

[12] Edman KAP, Caputo C (2017). Release of calcium into the myofibrillar space in response to active shortening of striated muscle. Acta Physiol..

[13] Stienen GJ (2015). Pathomechanisms in heart failure: The contractile connection. J. Muscle Res. Cell Motil..

[14] Biesiadecki BJ, Davis JP, Ziolo MT, Janssen PML (2014). Tri-modal regulation of cardiac muscle relaxation; intracellular calcium decline, thin filament deactivation, and cross-bridge cycling kinetics. Biophys. Rev..

[15] Leite-Moreira AF (2006). Current perspectives in diastolic dysfunction and diastolic heart failure. Heart.

[16] Zile MR, Brutsaert DL (2002). New concepts in diastolic dysfunction and diastolic heart failure: Part II: Causal mechanisms and treatment. Circulation.

[17] Tomofuji K (2015). Takotsubo cardiomyopathy with transient left ventricular obstruction successfully treated with cibenzoline succinate: A case report. J. Cardiol. Cases.

[18] Redfors B (2013). Stress-induced cardiomyopathy (Takotsubo): Broken heart and mind?. Vasc. Health Risk Manag..

[19] Nisa A (2021). Jellyfish venom proteins and their pharmacological potentials: A review. Int. J. Biol. Macromol..

[20] Shen ZD (2019). The urothelium enhancive polarization in CfTX-1 peptide intervened toad urinary bladder. Afr. J. Biotechnol..

[21] Faqihi F (2020). Reverse takotsubo cardiomyopathy in fulminant COVID-19 associated with cytokine release syndrome and resolution following therapeutic plasma exchange: A case-report. BMC Cardiovasc. Disord..

[22] Meyer P (2020). Coronavirus disease 2019 (COVID-19) and cardiac injury. JAMA Cardiol..

[23] Taza F, Zulty M, Kanwal A, Grove D (2020). Takotsubo cardiomyopathy triggered by SARS-CoV-2 infection in a critically ill patient. BMJ Case Rep..

[24] Chung MK (2021). COVID-19 and cardiovascular disease: From bench to bedside. Circ. Res..

[25] Abbott BC, Aubert XM (1952). The force exerted by active striated muscle during and after change of length. J. Physiol..

[26] Hill AV, Howarth JV (1959). The reversal of chemical reactions in contracting muscle during an applied stretch. Proc. R. Soc. Lond. B..

[27] Sugi H (1972). Tension changes during and after stretch in frog muscle fibres. J. Physiol..

[28] Edman KAP, Tsuchiya T (1996). Strain of passive elements during force enhancement by stretch in frog muscle fibres. J. Physiol..

[29] Herzog W, Leonard TR (2002). Force enhancement following stretching of skeletal muscle: A new mechanism. J. Exp. Biol..

[30] Herzog W, Lee EJ, Rassier DE (2006). Residual force enhancement in skeletal muscle. J. Physiol..

[31] Hisey B, Leonard TR, Herzog W (2009). Does residual force enhancement increase with increasing stretch magnitudes?. J. Biomech..

[32] Gibbs CL, Gibson WR (1970). Effect of alterations in the stimulus rate upon energy output, tension development and tension-time integral of cardiac muscle in rabbits. Circ. Res..

[33] Harikumar G (2009). Tension-time index as a predictor of extubation outcome in ventilated children. Am. J. Respir. Crit. Care Med..

[34] Horiuti K (1986). Some properties of the contractile system and sarcoplasmic reticulum of skinned slow fibres from *Xenopus* muscle. J. Physiol..

[35] Sewanan LR, Moore JR, Lehman W, Campbell SG (2016). Predicting effects of tropomyosin mutations on cardiac muscle contraction through myofilament modeling. Front. Physiol..

[36] Suga H (1987). Force-time integral decreases with ejection despite constant oxygen consumption and pressure-volume area in dog left ventricle. Circ. Res..

[37] Alpert NR, Blanchard EM, Mulieri LA (1989). Tension-independent heat in rabbit papillary muscle. J. Physiol..

[38] Powers JD (2020). Modulating the tension-time integral of the cardiac twitch prevents dilated cardiomyopathy in murine hearts. J.C.I. Insight..

[39] Hepple RT (2010). The O_2_ cost of the tension-time integral in isolated single myocytes during fatigue. Am. J. Physiol. Regul. Integr. Comp. Physiol..

[40] Brinkman DL (2014). *Chironex fleckeri* (box jellyfish) venom proteins: Expansion of a cnidarian toxin family that elicits variable cytolytic and cardiovascular effects. J. Biol. Chem..

[41] Andreosso A (2018). Structural characterisation of predicted helical regions in the *Chironex**fleckeri**CfTX-1* toxin. Mar. Drugs.

[42] Gomes HL (2016). Cardiovascular effects of Sp-CTx, a cytolysin from the scorpionfish (Scorpaenaplumieri) venom. Toxicon.

[43] Wu PL, Chiu CR, Huang WN, Wu WG (2012). The role of sulfatide lipid domains in the membrane pore-forming activity of cobra cardiotoxin. Biochim. Biophys. Acta..

[44] Wang Q, Zhang H, Wang B, Wang C, Xiao L, Zhang L (2017). β adrenergic receptor/cAMP/PKA signaling contributes to the intracellular Ca^2+^ release by tentacle extract from the jellyfish *Cyanea capillata*. BMC Pharmacol. Toxicol..

[45] Mustafa MR, White E, Hongo K, Othman I, Orchard CH (1995). The mechanism underlying the cardiotoxic effect of the toxin from the jellyfish *Chironex**fleckeri*. Toxicol. Appl. Pharmacol..

